# Endothelin as a Treatment Target in Cardiovascular Diseases: A Recent Step Forward

**DOI:** 10.31083/RCM45417

**Published:** 2025-12-22

**Authors:** Aleksandra Cole, Kajetan Kiełbowski, Aleksandra Dach, Jacek Szulc, Estera Bakinowska, Andrzej Pawlik

**Affiliations:** ^1^Department of Physiology, Pomeranian Medical University, 70-111 Szczecin, Poland

**Keywords:** cardiovascular diseases, endothelin-1, arterial hypertension, pulmonary hypertension

## Abstract

Cardiovascular diseases (CVDs) are a leading cause of mortality, significantly influencing quality of life and causing a burden on the healthcare system. Current treatment strategies utilize modern therapeutics, such as sodium–glucose cotransporter 2 (SGLT2) inhibitors and proprotein convertase subtilisin/kexin type 9 (PCSK9) inhibitors, which are both effective and safe. However, despite current medicines, acute cardiovascular events and chronic complications of CVDs remain significantly prevalent. Furthermore, CVDs are strongly linked to metabolic and inflammatory conditions that create a pathophysiological network of interactions, worsening the health of individuals. Therefore, identifying novel therapeutic targets and treatment combinations is of great importance to further mitigate the harmful effects of CVDs. Recently, aprocitentan, an endothelin-1 inhibitor, was approved to treat arterial hypertension. Meanwhile, endothelin has become a therapeutic target in CVDs, with inhibitors previously registered and used to treat pulmonary hypertension. Thus, this review aims to comprehensively discuss the role of endothelin-1 as a therapeutic target in CVDs and related disorders.

## 1. Introduction 

Cardiovascular diseases (CVDs) represent a broad group of disorders, such as atherosclerosis, ischemic heart disease, stroke, and arterial hypertension, which are associated with a significant global burden. Moreover, CVDs largely contribute to mortality, loss of health quality, and healthcare system costs. Meanwhile, the development of CVDs is strongly influenced by modifiable risk factors, including elevated blood pressure (BP), an unhealthy diet, metabolic alterations (e.g., high low-density lipoprotein (LDL) levels and high glucose concentrations), and low physical activity [1]. These disorders lead to vascular and organ remodeling, as well as inflammatory changes that eventually cause major cardiovascular adverse events. Hence, risk factor management and broad prevention represent key areas that are targeted to lower CVD prevalence. Nonetheless, the epidemiology of CVDs is concerning, as the occurrence of coronary heart disease, hypertension, stroke, and heart failure (HF) in patients over 20 years old is 48.6%, the majority of which is attributed to hypertension [2]. Currently, a vast array of therapeutics is available to manage CVDs, which reduce mortality and increase quality of life [3]. One of the key pathways involved in the pathophysiology of CVDs is the renin–angiotensin–aldosterone system (RAAS). Notably, angiotensin II (Ang II) is the primary factor contributing to hypertension and vascular and cardiac remodeling, which can lead to HF. Consequently, agents that suppress Ang II activity, such as angiotensin-converting enzyme inhibitors and Ang II receptor blockers, are widely used to treat patients with CVDs.

Recently, increased attention has focused on the endothelin (ET) system; specifically, ET-1, a 21-amino-acid peptide that plays a crucial role in regulating vascular tone. ET-1 functions as a vasoconstrictor and promotes the growth of vascular smooth muscle cells (VSMCs). Furthermore, ET-1 is involved in a network of interactions with other molecules that regulate vascular behavior, such as nitric oxide (NO), Ang II, and neuropeptide Y, among others, and the expression of ET-1 is regulated by hypoxia and shear stress. ET-1 signaling occurs through ET-A and ET-B, specific G protein-coupled receptors (GPCRs) that bind to associated ligands, thereby promoting the release of second messengers and an influx of calcium ions. Furthermore, GPCRs can stimulate tyrosine kinase pathways, such as the mitogen-activated protein kinase (MAPK), Src, phosphoinositide 3-kinase (PI3K), and Janus kinase (JAK) pathways, which are typically associated with corresponding effects on VSMCs [4]. ET-1 also exhibits a range of immunoregulatory properties, modulating both innate and adaptive immune responses [5]. Moreover, ET-1 is suggested to play a role in the pathogenesis of conditions including fibrosis [6], preeclampsia [7], and cancer [8, 9]. Additionally, dysregulation of ET receptors is associated with pathological processes; for example, renal pathologies increase activity of the ET-1/ET-A axis, promoting inflammatory responses, fibrosis, and hypertrophy [5].

In 2024, aprocitentan, an ET-1 antagonist, was approved for the treatment of resistant hypertension [10], thereby highlighting the ongoing importance of ET-1 inhibitors in CVDs. Therefore, this review aims to discuss the latest findings on the role of ET-1 in the pathophysiology of CVDs.

## 2. Endothelin and Cardiovascular Diseases: An Important Therapeutic Target

### 2.1 Endothelin Links to Essential Hypertension

Despite being largely preventable, hypertension, which is highly prevalent, represents an increasingly pressing social problem, contributing significantly to CVDs worldwide. Prolonged untreated hypertension affects the kidneys, brain, heart, and peripheral vasculature, and can cause disability and premature mortality. The pathogenesis of hypertension is complex, involving the dysregulation of multiple molecular and physiological pathways [11]. Meanwhile, advanced drug development has highlighted the therapeutic potential of targeting the endothelin system in the treatment of arterial hypertension.

Since the discovery of ET-1 in 1988, the molecule has been attributed vasoconstrictive properties. Indeed, ET-1 has been suggested to regulate intrinsic vascular tone and may contribute to the pathogenesis of hypertension [12]. Subsequent animal and human studies have demonstrated elevated plasma ET-1 levels in individuals with hypertension compared with normotensive controls. For instance, one of the key experimental studies in rats during the 1990s found an increase in both ET-1 levels and the basal release of ET-1 from mesenteric arteries during weeks 5 and 6 (the development of hypertension) in spontaneously hypertensive rats (SHRs) compared with age-matched controls. However, a concurrent study reported no difference in ET-1 plasma levels or arterial tissue expression between 6-week-old SHRs and control rats. Interestingly, a significant elevation in plasma, but not tissue, ET-1 levels was observed in week 16, thereby indicating enhanced ET-1 release in the later stages of disease progression [13]. In the context of human trials, data on the pathophysiological role of ET-1 in essential hypertension are limited. Intravenous administration of ET-1 to healthy subjects was shown to increase BP, which responded to a non-selective ET receptor antagonist [14]. Some authors have found that ET-1 levels were within the normal range in patients with essential hypertension, but increased ET-1 expression was detected in the vascular wall. The observed discrepancies in these results regarding circulating endothelin levels could result from the rapid elimination of ET-1 from the plasma, *i*.*e*., ET-1 has a plasma half-life of 1–2 min [15], and the directional secretion of ET-1 by endothelial cells (ECs) toward the VSMCs rather than into the circulation [16]. A recent study by Kostov and Blazhev [17] was designed to clarify whether ET-1 levels are elevated in essential hypertension. The researchers measured ET-1 alongside the associated precursor in 60 hypertensive and 20 normotensive patients. The results confirmed that patients in the hypertension group had significantly higher ET-1 levels than those in the control group, while the precursor levels were similar between groups [17]. However, personal factors, including comorbidities, race, salt intake, and specific instruments used to determine endothelin levels and their specificity, can also influence ET-1 concentrations [15].

#### 2.1.1 ET-1 Synthesis and Genetics in Hypertension

Many factors have been proven to stimulate ET-1 synthesis in both experimental and clinical studies, including shear stress, hypoxia, acidosis, cytokines (interleukin 1 (IL-1), IL-3, transforming growth factor β (TGF-β), interferon-γ (IFN-γ)), hormones (adrenalin, Ang II, vasopressin, cortisol), glucose, thrombin, and certain drugs. These stimuli are directly relevant to clinical conditions; for example, hypoxia is central to pulmonary hypertension, Ang II is a key factor in systemic hypertension, and thrombin reflects the link between coagulation and vascular tone. Thus, inhibition of the *Edn1* gene promoter, which encodes ET-1, occurs with well-known vasodilators, including NO, bradykinin, prostacyclin, atrial natriuretic peptide (ANP), and B-type natriuretic peptide (BNP) [18]. At the molecular level, transcription of *Edn1* is regulated by activator protein 1 (AP-1) and hypoxia-inducible factor-1 (HIF-1). Other enzymes known to generate ET-1 include mast cell chymase, matrix metalloproteinase-2 (MMP-2), and neutral endopeptidase; however, the functional significance of these enzymes remains underexplored and has no current therapeutic application [19]. The effect of shear stress on ET-1 production depends on the nature and intensity of the mechanical forces. Acute changes in mechanical stress transiently alter vessel diameter, primarily by releasing vasoactive mediators that help restore baseline vascular tone. In contrast, sustained mechanical changes lead to long-term remodeling of the vessel wall, involving alterations in the shape and cellular or extracellular composition of the wall. Vascular remodeling will be covered in more detail in other sections of this article. These biomechanical influences are clinically relevant, since disturbed flow and abnormal shear stress are central to the pathophysiology of many CVDs. The delicate interplay between shear stress and endothelial behavior is regulated by multiple signaling pathways. Endothelial cell proliferation, migration, and inflammatory response are mediated by the extracellular signal-regulated kinase (ERK), p38 MAP kinase (p38), c-Jun N-terminal kinase (JNK), and MAPK signaling pathways. Activation of endothelial nitric oxide synthase (eNOS) reduces inflammation, induces vasodilation, and inhibits platelet aggregation. Shear stress also modulates key transcription factors, including Krüppel-like factor 2 (KLF2) and nuclear factor kappa-light-chain-enhancer of activated B cells (NF-κB), which govern the expression of shear-sensitive genes.

Over the past 20 years, researchers have investigated the role of the *Edn1* gene as a potential risk factor for hypertension, yielding inconsistent results. Currently, these associations are considered exploratory rather than established risk factors. Furthermore, the variable impact of genetic polymorphisms on BP regulation may be influenced by environmental factors such as physical condition, obesity, or socioeconomic status [20]. Several studies have reported a positive correlation between the *ET-1 rs5370* T allele and BP and body mass index (BMI) across different populations, including European, Japanese [21], Australian, and American [22]. A recent study of the Malay ethnic group analyzed the *ET-1* (*rs5370*) and endothelin convertase enzyme (*ECE*) (*rs212526*) gene variants in 177 cases and 196 controls. The authors confirmed significant differences between the groups in both gene polymorphisms, suggesting that these variants may serve as potential genetic risk markers in this specific population [22].

Another single-nucleotide mutation, *rs9349379*, in the *PHACTR1* gene is associated with hypertension and increased *Edn1* gene expression. Interestingly, the link with hypertension is inverse [23]. The author suggested that the paradoxical reduction in hypertension, despite increased *Edn1* expression, may be explained by enhanced endothelin B-mediated vasodilation [19, 23]. *PHACTR1* has been shown to influence the cytoskeleton network, thereby altering arterial stiffness [24]. This single-nucleotide polymorphism (SNP) in the *PHACTR1* gene is of significant interest in precision medicine, as the SNP may help identify patients most likely to benefit from endothelin receptor antagonist therapy. This is due to increased active ET-1 signaling that needs to be blocked. In a study by Al Hageh *et al*. [25], based on a large UK Biobank cohort, the researchers found a genetic association between carriers of the *rs9349379*-G allele in the *PHACTR1* gene and an increased risk of developing severe or multivessel disease. In contrast, the *rs445925*-T variant near the *APOC1*/*APOE* locus is associated with a reduced risk of severe and multi-vessel coronary artery disease [25]. A recent study also identified endothelial *PHACTR1* as a novel corepressor of peroxisome proliferator-activated receptor gamma (PPARγ), promoting atherosclerosis in regions exposed to disturbed flow [26]. These findings highlight *PHACTR1* as a potential therapeutic target and support the use of genetic profiling to improve risk stratification and develop individualized therapies.

Genome-wide association studies have identified the Jumonji domain-containing protein D3 (JMJD3) locus, also known as lysine demethylase 6B (*KDM6B*), which encodes the histone demethylase JMJD3, as a major player in regulating systolic BP [27]. Histone demethylase encodes for a macrophage phenotype switch in cardiometabolic diseases, modulating inflammatory gene expression. Importantly, JMJD3 exerts context-specific effects depending on the cell type: JMJD3 promotes proinflammatory processes in macrophages, whereas JMJD3 supports vascular homeostasis in VSMCs. A recent study investigated epigenetic alterations in VSMCs during hypertensive arterial remodeling, underscoring the role of JMJD3 in maintaining ET-1 receptor balance and preventing a vascular phenotypic switch. The authors have highlighted the *JMJD3 rs62059712* variant as the regulator of JMJD3 expression in VSMCs. Moreover, JMJD3 enhances ET-B expression, and, in the absence of the gene, increased ET-A transcription is observed as a compensatory effect. The major *rs62059712*-T allele decreases *JMJD3* transcription, and JMJD3 loss is associated with upregulated endothelin signaling, resulting in subsequent HTN-induced arterial remodeling [28]. A separate study investigating vascular remodeling in abdominal aortic aneurysms has shown that JMJD3 in macrophages is upregulated by IFN-β, leading to NF-κB-mediated overexpression of inflammatory genes [29]. Although there is no direct evidence that JMJD3 amplifies ET-1 signaling through NF-κB in the hypertension model, it is reasonable to hypothesize that JMJD3 may indirectly contribute to inflammation via this pathway. NF-κB has been shown to upregulate *Edn1* transcriptionally, increasing ET-1 levels in vascular cells under inflammatory conditions. Conversely, ET-1, acting through ET-A and ET-B receptors, is known to activate NF-κB pathways, leading to the expression of proinflammatory cytokines and adhesion molecules that facilitate monocyte/macrophage infiltration. Therefore, JMJD3 may amplify ET-1 signaling both directly, via NF-κB activation, and indirectly, by sustaining macrophage-driven vascular wall inflammation [30]. Although the literature discusses the genetic aspects of ET-1, the clinical relevance of these studies on ET-1 is not widely applicable.

#### 2.1.2 ET-1-Induced Vasoconstriction 

In a healthy organism, the vasoconstrictor effects of ET-1 play an important role in maintaining vascular tone via the ET-A receptor on VSMCs. ET-A receptors are abundant in the human body and have been well-documented in the cerebral vasculature, coronary vascular network, and cardiomyocytes, as well as the pulmonary artery and various components of the kidney, including the afferent arteriole, efferent arteriole, cortical vessels, mesangial cells, and renal artery [31, 32]. The vasoconstrictive pathway is believed to involve G protein-coupled signaling. Evidence indicates that both ET-A and ET-B receptors can interact with different G protein subtypes, including Gq, Gi, and G12/13, leading to diverse intracellular signaling cascades. This diversity may potentially explain the wide range of physiological and pathological effects mediated by ET-1, including the complex role performed by ET-1 in hypertension. Receptor stimulation by ET-1 results in phospholipase C (PLC) hydrolyzing phosphatidylinositol biphosphate (PIP2) to inositol 1,4,5-triphosphate (IP3) and diacylglycerol (DAG). IP3 increases intracellular Ca^2+^ levels while DAG stimulates protein kinase C (PKC), which promotes VSMC contractions.

Current scientific literature does not provide evidence that ET-A receptor activation directly causes vasodilation. This role is attributed to the ET-B receptor present on ECs. In contrast to the ET-A receptor, the ET-B receptor is expressed on both VSMCs and ECs, but the function of the ET-B receptor differs for each. The interaction between ET-1 and ET-B can indirectly promote vasodilation due to the activity of NO and prostaglandin I2 (PGI2), whose synthesis is stimulated by intracellular Ca^2+^. ET-1 and Ang II share a common architecture of receptor-mediated signaling with two arms: the first, the dominant vasoconstrictive/pro-fibrotic arm, and the second, the protective vasodilatory arm (ET-B and AT2R). Ang II exerts primary physiological effects through the Ang II type 1 receptor (AT1R). Upon activation, the AT1R primarily signals through the Gq/11 protein pathway, leading to vasoconstriction. Additionally, AT1R stimulation promotes VSMC proliferation, hypertrophy, and migration. These conditions contribute to pulmonary vascular remodeling and, ultimately, to right ventricular (RV) dysfunction, as seen in pulmonary hypertension [33].

In addition to the IP3/DAG pathway, ET-1 and Ang II are recognized activators of the MAPK signaling pathway, especially ERK1/2, which are tightly linked to vascular remodeling. Recently, researchers have identified that GPCR kinase (GRK2) fine-tunes the signaling cascade that mediates vasoconstriction. GRK2-facilitated GPCR phosphorylation can result in either reduced signaling through desensitization or prolonged signaling via β-arrestins. The group of receptors remaining under the influence of GRK2 includes ET-A and ATR1. It is worth noting that GRK2 expression is elevated in hypertension in both rat models and human patients [34].

#### 2.1.3 ET-1 in Endothelial Dysfunction

The binding of ET-1 to its receptors on VSMCs can activate both proinflammatory and pro-fibrotic processes, contributing to endothelial impairment. The term “endothelial dysfunction” encompasses several pathological conditions and broadly refers to a disturbance of the homeostatic role of the endothelium in regulating vasodilation and vasoconstriction, as well as inflammatory and anticoagulant processes [35].

Vascular inflammation is a major contributor to endothelial dysfunction and CVDs. ET-1 plays a pivotal role in endothelial cell activation, which includes the secretion of monocyte chemoattractant Protein-1 (MCP-1) after binding to ET-A and ET-B receptors, often via the NF-κB and MAPK signaling pathways. MCP-1 binds to the C-C chemokine receptor type 2 (CCR2) on circulating monocytes. This interaction promotes chemotaxis, causing monocytes to migrate toward the inflamed endothelium. Once inside the vessel, monocytes differentiate into macrophages, producing proinflammatory cytokines and oxidative enzymes [36]. Simultaneously, injured ECs secrete various inflammatory mediators and tissue factor (TF), amplifying both the local inflammatory response and coagulation cascade. Toll-like receptors (TLRs) are key mediators of this cascade, as are the receptor for advanced glycation end (RAGE) products and NOD-, LRR- and pyrin domain-containing protein 3 (NLRP3) inflammasome activation. Fais *et al*. [37] demonstrated that ET-1 activates the NLRP3 inflammasome through the calcium-dependent generation of reactive oxygen species (ROS). ET-1 involvement in endothelial dysfunction, alongside these aforementioned mechanisms, is thought to alter the function of various populations of K^+^ channels. These are inward-rectifying (Kir), ATP-sensitive (KATP), voltage-gated (Kv) and KCa channels. Among the KCa (Ca^2+^-activated K^+^ channels), KCa2.3 is essential for NO signaling in the vascular bed. Meanwhile, the expression or function of KCa2.3 channels is suppressed in arterial hypertension. Moreover, KCa2.3 activation exerts an anti-inflammatory effect and lowers BP. ET-1-mediated membrane conductance in ECs is suggested to result primarily from KCa2.2/2.3 inhibition [37].

Endothelial dysfunction is a key contributor to and consequence of hypertension through reciprocal exacerbation. Impaired endothelial function promotes increased vascular tone and inflammation, which, together with shear stress and mechanical injury, accelerates vascular damage. Although current evidence does not clearly establish which process initiates this cycle, the interplay between these processes ultimately promotes damage to multiple organs [38]. A retrospective cohort study on 456 patients with essential hypertension showed that endothelial dysfunction was a significant risk factor in developing subclinical target organ damage (STOD). Additionally, early improvement in endothelial function was an independent protective factor against STOD in patients, regardless of the baseline endothelial status of the patient [39].

The endothelium is known to self-regulate in an autocrine, paracrine, and endocrine fashion by secreting vasoconstrictive, prothrombotic, and proliferative substances, including ET-1, Thromboxane A2, ROS, and their opposite effectors, e.g., NO and prostacyclin. Moreover, ECs secrete multiple inflammatory markers that stimulate blood vessel contraction [40]. The primary ET-1-related contributors to endothelial dysfunction include an imbalance in vasoactive substances, heightened inflammation, excessive ROS production, and reduced NO synthesis (Table 1; Ref. [41, 42, 43, 44, 45, 46, 47, 48, 49, 50]).

**Table 1.** **Processes associated with endothelial dysfunction**.

Category	Main factors contributing to the pathology	Effects on the endothelium	Reference
Vascular resistance	Direct ET-1 effect on VSMCs	Increased tone/vasoconstriction	[41, 42]
	ET-1 decreased eNOS expression	Impaired vasodilation	
Oxidative stress	ET-1 binding to ET-A/ET-B stimulates NADPH oxidase activity and ROS release alongside eNOS uncoupling	Increased ROS, NO degradation, transcription, and activation of MMP-2, MMP-9, and extracellular matrix degradation	[43, 44]
		Further decrease in NO bioavailability	
	Hydrogen peroxide	Endothelial apoptosis	
	Superoxide anion	Inflammation induction	
G-protein coupled signaling ET-1 coupled with Gq, Gi, G12-13	PLC, MAPK, RhoA pathways	Altered vascular tone and permeability	[45, 46]
Inflammatory response	Cytokine release (IL-6, TNF-α)	Recruitment of immune cells promoting chronic vascular inflammation	[47]
	NF-κB pathway	Upregulation of inflammatory gene expression in macrophages	[48]
	COX (mainly COX-2) upregulation	Increased PGE_2_, PGF_2⁢α_ expression, decreased PGI_2_ resulting in vasoconstriction	[49]
Vascular remodeling	Initiated by increased KLF4 expression induced by ET-1	Contractile to synthetic phenotypic switch	[50]
	ET-1 activates macrophages to remodel the ECM through TGF, IL-13, and IL-10	Collagen deposition/fibrosis	

NADPH, the reduced form of nicotinamide adenine dinucleotide phosphate; IL, interleukin; TNF-α, tumor necrosis factor-α; NF-κB, nuclear factor kappa-light-chain-enhancer of activated B cells; COX, cyclooxygenase; COX-2, cyclooxygenase-2; PGE_2_, prostaglandin E2; PGF_2⁢α_, prostaglandin F2α; PGI2, prostaglandin I2; KLF4, Krüppel-like factor 4; TGF, transforming-growth factor family; MMP, matrix metalloproteinase.

Another area linking ET-1 and endothelial dysfunction is the association with lectin-like oxidized LDL receptor 1 (LOX-1). This receptor is known to be involved in endothelial dysfunction within the context of atherosclerosis. The receptor binds oxidized LDL, promotes the expression of adhesion molecules, and disrupts endothelial vasodilation. Moreover, this interaction disrupts NO signaling and promotes ROS release. Additionally, LOX-1 promotes ET-1 production [51]. Intriguingly, an early study confirmed that ET-1 upregulates LOX-1 expression and promotes oxLDL uptake in ECs [52].

ROS are important signaling molecules through which vasoactive substances, such as ET-1, Ang II, and proteinoids, mediate cellular effects [53]. Under physiological conditions, the majority of the produced NO is facilitated by eNOS, making this enzyme critical for effective vascular regulation and cardiovascular protection [43]. Indeed, eNOS and the reduced form of nicotinamide adenine dinucleotide phosphate (NADPH) oxidase play opposing but interconnected roles. The imbalance between NO and ROS, resulting, for example, from NADPH oxidase overactivity, leads to NO degradation and eNOS uncoupling, which further promotes ROS release. Recent studies using eNOS-deficient murine models have shown that the absence of eNOS results in sustained elevation of BP and alterations in vascular structure associated with endothelial dysfunction, including a reduced inner diameter in the descending thoracic aorta and increased wall thickness [54]. Since ET-1 has been shown to suppress eNOS function, the expected effects are reduced NO availability and exacerbated endothelial dysfunction. Meanwhile, xanthine oxidase (XO) represents another source of ROS in hypertension, which has been historically suggested and trialed as a target therapeutic approach in hypertension [43]; however, the results were inconsistent, and there were adverse effects associated with the treatment [55]. ECs function as sensitive mechanoreceptors, constantly adapting to the physical forces generated by blood flow. Additionally, laminar shear stress (LS) predominates in regions where blood flows smoothly and in a defined direction, such as straight arterial segments. This form of flow promotes endothelial stability and vascular homeostasis. In contrast, oscillatory shear stress (OS) arises at arterial branches and curves due to disturbed flow. This non-uniform shear pattern fosters a proinflammatory state and is strongly linked to the focal development and progression of atherosclerotic plaques. Importantly, OS leads to upregulation of ET-1 via activation of diverse signaling cascades involving adaptor proteins, receptors, transcription factors, kinases, junctional proteins, and adhesion molecules [56].

In recent years, short non-coding RNAs, also known as microRNAs (miRNAs), have gained significant attention as key modulators of vascular function, particularly in hypertension and arterial remodeling. One of the most significant findings is that miRNAs mediate internal communication between ECs and VSMCs. Meanwhile, miRNAs are often packaged into extracellular vesicles, such as microvesicles or exosomes, and released into the circulation. Interestingly, encapsulation makes the miRNAs resistant to degradation. Among the studied miRNAs, miR-92a emerged as a critical regulator of endothelial homeostasis. A recent study found that miR-92a levels were higher in hypertensive patients than in controls. Moreover, the miR-92a levels were positively correlated with systolic and diastolic BPs and ET-1 levels, and negatively correlated with NO levels. Ang II has been shown to increase miR-92a expression in ECs, which, in turn, mediates increased arterial stiffness [57]. It has been documented that miRNAs participate in the macrophage phenotype switch and atherogenesis, including miR-19, miR-21, miR-33, miR-150, miR-223, and let-7c. Several miRNAs have been identified that directly or indirectly influence ET-1 expression or signaling. These include miR-125a, miR-125b, miR-199a, miR-155, miR-1, and miR-320, which downregulate ET-1 by targeting *Edn1* in most cases. Moreover, some of these miRNAs can exert the opposite effect by indirectly upregulating ET-1, as seen with miR-21 [58]. Recent research has highlighted that miR-33 exhibits the strongest upregulation in expression in exosomes derived from human umbilical vein endothelial cells (HUVECs) after stimulation with ET-1. Furthermore, the authors observed that nuclear receptor subfamily 4, group A (NR4A) has a binding site for miR-33 in the 3^′^ untranslated region (UTR), emphasizing the existence of the miR-33/NR4A axis. Meanwhile, miR-33 inhibition downregulated proinflammatory macrophage genes (*IL-6*, *iNOS*, and *TNF-α*). ET-1 knockdown inhibited proinflammatory macrophage activation. Collectively, these findings suggest that the miR-33/NR4A axis is required for ET-1-induced proinflammatory macrophage activation [59].

#### 2.1.4 The Role of ET-1 in Vascular Remodeling

The ET-1-induced signaling pathway generates ROS in parallel to Ca^2+^ liberation and smooth muscle contraction. ROS are the product of multiple enzymatic sources, including NADPH oxidase, xanthine oxidase, uncoupled eNOS, and mitochondria, in response to growth factor receptor activation. Experimental animal and *in vitro* models suggest that vascular remodeling involves the activation of c-Src (a non-receptor tyrosine kinase) and MAPK cascades, which contribute to cellular proliferation and, ultimately, structural changes in the vessel wall [60]. Remodeling processes are influenced by hemodynamic forces. Diffuse intimal thickening appears to be an adaptive structural response to altered mechanical forces, typically occurring in vascular regions characterized by reduced wall shear stress, increased wall tensile stress, or a combination of both. A recent study has highlighted the remarkable plasticity of VSMCs under pathological conditions. VSMCs can undergo phenotypic switching from their native contractile state to various other phenotypes as a compensatory mechanism in hypertension-driven maladaptive remodeling [61]. The loss of TGF-β in upstream signaling, particularly when combined with hyperlipidemia, can trigger the phenotypic switch in VSMCs. In animal models, the major factor driving the transition was shown to be an approximately 100-fold upregulation of Krüppel-like factor 4 (*KLF4*) and other KLF family members [50]. The interaction between the KLF family and the endothelin system has been well documented experimentally [62, 63]. The KLF transcription factor, expressed in the ECs, exerts anti-inflammatory and vasoprotective effects by increasing eNOS and thrombomodulin and suppressing *Edn1* gene expression. Conversely, ET-1 has been shown to increase *KLF4* gene expression, potentially establishing a feedback mechanism that contributes to vascular homeostasis [64]. The role of KLF4 in the unique population of adventitial progenitor cells was also studied, revealing that KLF4 contributes to preventing stem cell differentiation into VSMCs, normalizes the adventitia-to-media ratio, and mediates collagen deposition. Thus, the ET-1/KLF4 self-regulatory cycle may profoundly influence vascular homeostasis [65]. Overall, the direct application of these findings to humans remains uncertain.

Essential hypertension is characterized by increased peripheral vascular resistance, primarily due to functional changes in small, resistant arteries. Vasodilation in these vessels is mainly mediated by NO, as evidenced by the inhibition of vasodilation through eNOS blockers, with additional support from NO-independent mechanisms such as endothelium-dependent hyperpolarization (EDHF). Notably, EDHF assumes a compensatory vasodilatory role when NO bioavailability is low. Thus, remodeling induced by cardiovascular risk factors results in reduced vasodilation in response to endothelial agonists, such as acetylcholine. A recent study on the relationship between resistant artery remodeling, NO availability, and endothelial function demonstrated that a progressive deficit in NO availability may be a more accurate measure of various stages of small resistance artery remodeling than traditional hemodynamic measures, such as hypertension severity. However, the study did not investigate the potential interaction between NO availability and other pathways regulating resistance vessel tone, such as the ET-1 or EDHF pathways. These pathways could potentially have an equally important diagnostic role; however, further research is needed to describe these dynamics in resistance vessel remodeling [66].

ET-1-induced oxidative stress in vascular remodeling is primarily mediated by ROS, which activate a family of proteolytic MMP enzymes. Hyperactivity of MMPs results in the degradation of extracellular matrix components. As a result of their activity, basement membrane and intracellular junctions are degraded, along with small proteins such as cell adhesion molecules, peptide growth factors, cytokines, chemokines, and tyrosine kinase receptors. MMP-2 and MMP-9 are important in vascular remodeling; the activity of these proteins is associated with both the early and late phases of hypertension [43].

#### 2.1.5 Link to Salt-Sensitive Hypertension

High dietary salt intake can induce BP changes even in normotensive individuals, with approximately 26% demonstrating a salt-sensitive response. Among hypertensive patients, this proportion is even higher, with about 51% exhibiting BP elevations in response to high salt intake [67]. Salt-sensitive hypertension is often associated with impaired natriuresis, endothelial dysfunction, and heightened inflammatory responses. In this context, recent findings from a transgenic mouse model provide important mechanistic insights. Mice with selective myeloid ET-B receptor knockout and Ang II-induced hypertension were assessed for BP and end-organ damage (kidneys, eyes, cardiovascular). While the ET-B receptor in the kidneys facilitates sodium excretion, ET-B signaling in myeloid cells appears to promote inflammation and vascular damage during hypertension. Surprisingly, the deletion of ET-B in these immune cells led to reduced hypertensive organ injury and improved endothelial function, thereby lowering BP over time. These findings suggest that the immune–endothelin axis plays a role in modulating salt-sensitive responses [68]. Interestingly, the selective deletion of ET-1 in the collecting duct of the nephron has been shown to elevate BP and to develop a salt-sensitive hypertensive phenotype. Similar outcomes were observed following the targeted deletion of ET-B receptors or both ET-A and ET-B receptors in the collecting duct [15]. Furthermore, the cross-interactions between ET-1 and the RAAS amplify the pressor response: Ang II stimulates ET-1 production, while ET-1 enhances the conversion of Ang I to Ang II. Both pathways independently promote the release of aldosterone, another potent vasoconstrictor, thereby creating a self-reinforcing cycle that contributes to the pathogenesis of salt-sensitive hypertension [69]. Becker *et al*. [70] found that ET-B receptor activation during high-salt intake impaired baroreflex sensitivity and increased BP variability, key features in salt-sensitive hypertension. Additionally, ET-B receptors in the kidney, when stimulated by ET-1, normally promote natriuresis; thus, dysfunction in this mechanism contributes to volume-dependent hypertension. In salt-resistant hypertension, ET-1 contributes more prominently through sustained vasoconstriction and structural vascular changes [70].

#### 2.1.6 ET-1 Inhibitors in Arterial Hypertension

Until recently, the use of ERAs has been nearly exclusively constrained to the treatment of pulmonary arterial hypertension. However, due to the potent vasoconstrictive properties of the endothelin system, these agents offer a promising approach for treating resistant hypertension. According to the American Heart Association and the American College of Cardiology, resistant hypertension is diagnosed when three different classes of antihypertensive drugs have been applied concurrently, yielding no improvement in hypertensive status [71]. The PRECISION clinical trial investigated the potential use of a dual ET-A/ET-B endothelin receptor antagonist in patients with resistant hypertension. In the initial 4-week double-anonymized phase, patients receiving aprocitentan once daily experienced reductions in office systolic BP of 15.3 mmHg and 15.2 mmHg, respectively, compared to an 11.5 mmHg reduction with placebo. This corresponds to placebo-adjusted reductions of 3.8 mmHg and 3.7 mmHg for the 12 mg and 25 mg doses, respectively. These findings were corroborated by 24-hour ambulatory BP monitoring, which showed reductions of 4.2 mmHg and 5.9 mmHg for the mentioned doses, respectively. The BP-lowering effect of aprocitentan was sustained over the 32-week single-anonymized phase [72, 73].

The progression of CVDs can be partially attenuated through the management of cardiovascular risk factors and the use of pharmacological agents targeting various pathways, including ERAs, RAAS inhibitors, and glucose-lowering therapies—all of which have been shown to enhance vascular health; combinations of ET-1 inhibitors with other pharmacological groups are being examined. A subgroup analysis of the PRECISION trial, which involved aprocitentan and a sodium–glucose cotransporter 2 (SGLT2) inhibitor in patients with diabetes, was presented at an American College of Cardiology meeting. Systolic BP reductions by week 36 were comparable between the aprocitentan+SGLT2 and aprocitentan only groups (11.8 mmHg and 13.8 mmHg respectively). The impact on urinary albumin–creatinin ratio was greater when aprocitentan was combined with the SGLT2 inhibitor. The data support that aprocitentan remains effective in reducing systolic BP, whether or not SGLT2 inhibitors are co-administered [74].

**Box 1.** **Overview of endothelin-1**.

∙ Endothelin-1 is a peptide with primary vasoconstrictive properties.
∙ Endothelin-1 regulates intracellular calcium levels and influences the activity of signaling cascades.
∙ Endothelin-1 signals through ET-A and ET-B receptors, which stimulate different responses.
∙ Endothelin-1 stimulate proinflammatory and pro-fibrotic processes.
∙ Inhibition of endothelin-1, a well-known strategy in the treatment of pulmonary hypertension, was recently approved to treat arterial hypertension.

### 2.2 ET-1 Inhibitors in Renal Diseases

Hypertension is both a cause and a consequence of nephropathy, characterized by podocyte loss, glomerular injury, and mesangial expansion. Many hypertensive patients—especially those with diabetes—still progress to end-stage renal disease despite controlling BP. In a diabetic mouse model of advanced nephropathy (BTBR ob/ob), combined treatment with the ET-A receptor antagonist atrasentan and the AT-1 receptor antagonist losartan significantly reduced proteinuria and increased the number of glomerular podocytes, suggesting that podocyte restoration likely occurs through parietal epithelial cells. Atrasentan alone also showed beneficial effects, though to a lesser extent. Both treatments reduced mesangial matrix expansion, as evidenced by decreased collagen type IV accumulation. This study supports the notion that combination therapies targeting multiple pathogenic pathways, such as RAAS and ET-1 in this case, are becoming increasingly rational [75]. Another study of combined treatment with sparsentan—a dual Ang II type 1 and ET-A antagonist (AT-1/ET-A) inhibitor in a single molecule under clinical development—was tested in a murine model of Alport syndrome. Sparsentan significantly improved kidney function, with 5 weeks of treatment leading to a GFR comparable to that of wild-type mice. Several proinflammatory and pro-fibrotic genes were downregulated (e.g., *Ccl2*, *Ccr2*) as were signaling genes (*Serpine1*, *Timp1*, *Tnxb*, *Myc*). Sparsentan treatment extended lifespan, even when initiated in mice with renal pathology (week 4), whereas losartan was less effective and delayed the onset of proteinuria compared with controls. In contrast, losartan-treated mice only showed a significant reduction in proteinuria at 8 weeks [76]. Sparsentan is currently approved to slow the progression of kidney function decline in adults with IgA nephropathy. The New Drug Application (sNDA) is supported by data from two pivotal studies: the Phase 3 DUPLEX study and the Phase 2 DUET study. Both studies demonstrated that sparsentan led to a rapid, sustained, and significantly greater reduction in proteinuria compared to the maximum labeled dose of irbesartan [77]. There are ongoing interventional studies on patients with focal segmental glomerulosclerosis (FSGS) and sparsentan. The drug has potential use in CVDs due to its ability to reduce proteinuria and BP.

Elevated glucagon and amino acids in the kidney promote afferent arteriolar vasodilation, while Ang II and ET-1 increase efferent arteriolar resistance, together raising glomerular pressure. Persistent hyperglycemia further exacerbates this pressure by enhancing SGLT2-mediated sodium and glucose reabsorption, reducing solute delivery to the macula densa, and blunting tubuloglomerular feedback. The resulting imbalance leads to glomerular hyperperfusion and hyperfiltration [78]. SGLT2 inhibitors provide cardiovascular and renal protection largely independent of their glucose-lowering effects [79], primarily by reducing glomerular hyperfiltration and improving tubuloglomerular feedback by increasing solute delivery (sodium, chloride, glucose) into the distal part of the nephron [80]. Additional benefits may arise from the anti-inflammatory, antioxidant, and hemodynamic effects of these inhibitors [81]. These complementary mechanisms support the rationale for combining SGLT2 inhibitors with ERAs to enhance outcomes in patients with cardio–renal disease. Meanwhile, further studies are required despite the initial outcomes of the subgroup in the PRECISION trial. Table 2 (Ref. [72, 82, 83, 84, 85, 86, 87]) lists the currently approved ET-1 inhibitors.

**Table 2.** **A summary of approved ET-1 inhibitors**.

Drug	Year of approval	Mechanism of action	Registered indication	Adverse events	References
Bosentan	FDA 2001	ET-A/ET-B antagonist	PAH	Abnormal liver function, severe anemia, and teratogenicity	[82]
	EMA 2002				
Sitaxentan	2006	ET-A antagonist	PAH	Liver failure (was subsequently withdrawn)	[83]
Ambrisentan	FDA 2007	ET-A antagonist	PAH	Headache, nasal congestion, and peripheral edema	[84]
	EMA 2008				
Macitentan	FDA 2013	ET-A/ET-B antagonist	PAH	Anemia, naso-pharyngitis, and headache	[85]
	EMA 2013				
Aprocitentan	2022	ET-A/ET-B antagonist	Resistant hypertension	Peripheral edema/fluid retention, anemia, headache	[72]
Clazosentan	Japan 2022	ET-A antagonist	Prevention of cerebrovascular spasm after surgery for subarachnoid hemorrhage caused by cerebral aneurysm and subsequent cerebral infarction and cerebral ischemic episodes	Headache, nausea, and nasal obstruction	[86]
Sparsentan	FDA 2023	ET-A/AT1R antagonist	IgA nephropathy	Hyperkalemia, elevated liver enzymes, and hepatotoxicity	[87]
	EMA 2024				

### 2.3 Atherosclerosis

Atherosclerosis is a condition characterized by the deposition of lipid and fibrous material in the intima, leading to the formation of an atheroma. The atherosclerotic plaque progressively becomes calcified and fibrous. Hence, as the plaque grows, arterial occlusion occurs and, consequently, tissue ischemia. Moreover, atherosclerotic plaque disruption can form a thrombus and, therefore, cause cardiovascular events such as stroke and acute coronary syndromes. The chronic presentations of atherosclerosis include stable angina, vascular dementia, non-ruptured aortic aneurysm, chronic limb ischemia, and chronic mesenteric ischemia [88]. In 2020, an estimated 21.2% of people aged 30–79 years had atherosclerotic plaques in the carotid artery [89]. In the Miami Heart Study on the U.S. population with a mean age of 53 years, the prevalence of coronary artery plaques was 49%. In contrast, in the SCAPIS study on the Swedish population aged 50–64, the prevalence of coronary atherosclerosis was 42.1% [90, 91]. The onset of chronic and acute manifestations of atherosclerotic cardiovascular disease (ASCVD) can be avoided by lifestyle changes and pharmacotherapy. Currently, most anti-atherosclerotic drugs aim to lower LDL plasma concentrations. According to ESC/EAS guidelines, statins are the first-line drugs for lowering LDL. Meanwhile, ezetimibe should be added when the maximum tolerated statin dose is reached and the therapeutic goal remains unmet. Furthermore, the addition of a PCSK9 inhibitor is recommended if the therapeutic goal is not achieved with the two-drug combination [92]. Inflammation is another therapeutic target that can be addressed by anti-atherosclerotic drugs. For example, low-dose colchicine treatment has been shown to reduce the risk of cardiovascular events, leading to the approval of colchicine use for patients with ASCVD by the United States Food and Drug Administration [93]. Moreover, antiplatelet drugs can be used in the secondary prevention of cardiovascular events [88].

The development of atherosclerosis begins with endothelial cell dysfunction, which is facilitated by factors such as hypercholesterolemia, hypertension, diabetes, disturbed blood flow, and oxidative stress [94]. Endothelial dysfunction is generally characterized by a change in its phenotype toward vasoconstrictive, prothrombotic, and proinflammatory [35]. LDLs that accumulate in the intima undergo oxidation; oxidized LDL is a key trigger of EC activation [95]. Activated ECs overexpress membrane-associated and secreted chemokines, prothrombotic molecules, and adhesion molecules, facilitating the entry of monocytes and T lymphocytes into the subendothelial space and further promoting local inflammation [94]. Monocytes and VSMCs accumulate cholesterol, which leads to the formation of foam cells and, together with extracellular cholesterol deposition, contributes to the development of atherosclerotic lesions. Further steps in plaque development include the formation of a necrotic core and fibrous cap and eventually plaque calcification [96].

Several studies investigated the role of ET-1 in the development of atherosclerosis. Zhang *et al*. [97] demonstrated that ET-1, via the ET-A receptor (which is absent in non-dysfunctional ECs), increases the production of chemokines and adhesion molecules by activated ECs in HUVECs and mouse models of atherogenesis. This facilitates monocyte migration and promotes the proinflammatory M1 macrophage phenotype, both of which contribute to the progression of atherosclerosis [97, 98]. In a subsequent *in vitro* study, Zhang *et al*. [59] showed that exosomal non-coding RNA is engaged in the ET-1-induced interaction between ECs and macrophages. ECs stimulated by ET-1 release exosomes containing miR-33, which downregulate NR4A in macrophages and, therefore, promote the M1 phenotype, which facilitates atherogenesis [59]. Arginase II is an enzyme that plays a role in atherosclerosis progression by promoting endothelial dysfunction by decreasing NO bioavailability and increasing oxidative stress. Arginase II also promotes ROS production in macrophages, thereby stimulating inflammatory responses [99, 100]. Rafnsson *et al*. [101] evaluated the influence of ET-1 on arginase in atherosclerosis. ET-1 was shown to upregulate arginase II in ECs. Moreover, the ET-1/ET-B axis promotes arginase II activity in macrophages, thereby enhancing ROS production [101]. NADPH oxidase 1 (NOX1) is another enzyme that contributes to atherosclerosis development by producing ROS [102]. Ouerd *et al*. [103] demonstrated that endothelium-specific overexpression of ET-1 accelerates diabetes-induced atherosclerosis by increasing NOX1 expression in a murine model; moreover, ET-1 overexpression reduced atherosclerotic plaque stability [103]. In line with this finding, Płoński *et al*. [104] showed that plasma ET-1 levels were significantly higher in patients with unstable carotid atherosclerotic plaques than in those with stable plaques. Furthermore, ET-1 mediates age-associated vascular stiffness by promoting fibrosis and the aforementioned effects on vascular dysfunction [15].

**Fig. 1.**
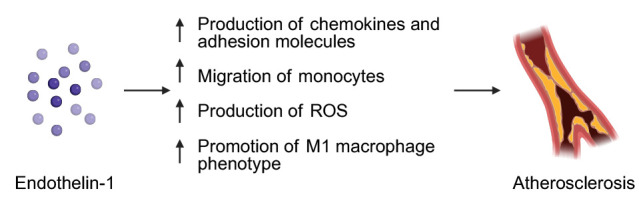
**The potential role of endothelin-1 in the pathogenesis of atherosclerosis**. Created in BioRender. Physiology, D. (2025) https://BioRender.com/2gwupoq.

Since ET-1 plays a role in atherosclerosis development, several studies have evaluated the potential use of both selective and non-selective endothelin receptor antagonists in the treatment of atherosclerosis. To begin with, atrasentan, an ET-A-selective antagonist, was shown to ameliorate the development of early atherosclerosis. After 6 months, significant reductions in normalized percent atheroma volume (PAV) and mean total atheroma volume (TAV) in coronary artery segments with endothelial dysfunction were observed in the atrasentan group [105]. In addition, in an earlier study, atrasentan was reported to improve endothelial function in patients with incipient atherosclerosis, as reflected by increased endothelium-dependent vasodilatation compared with placebo [106]. Houde *et al*. [107] showed that treatment with macitentan, a non-selective endothelin receptor antagonist, attenuates the development of atherosclerotic lesions and improves the plaque stability in ApoE knockout mice. Moreover, the authors evaluated the effect of deleting mouse mast cell protease 4 (mMCP-4), a murine analog of human chymase, on atherosclerosis [107]. Chymase is a mast cell-associated enzyme that is engaged in the first stage of an alternative pathway of ET-1 synthesis. First, chymase converts big ET-1 to ET-1 (1–31), which is then converted by neprilysin to ET-1 (1–21) [108]. Therefore, inhibition of this pathway is a potential alternative method of reducing ET-1 signaling and its consequences in human disease. In the study by Houde and colleagues [107], mMCP-4 knockout mice exhibited delayed progression of early atherosclerosis and greater plaque stability compared with controls. Xu *et al*. [109] demonstrated that another non-selective endothelin receptor antagonist, bosentan, attenuates the development of atherosclerosis in ApoE knockout mice. Moreover, bosentan exerted an anti-apoptotic effect on endothelial cells. The putative mechanism behind this effect was downregulation of programmed cell death protein 4 (PDCD4) via miRNA-21 [109]. PDCD4 is a protein known to facilitate atherosclerosis development by increasing apoptosis, autophagy, and inflammation [110]. A recent study corroborated the anti-atherosclerotic effect of bosentan treatment in ApoE knockout diabetic mice, as reflected by significantly reduced lumen stenosis in the bosentan-treated group compared with the control group (19.5 ± 2.2% versus 24.6 ± 4.8%; *p* < 0.05). Furthermore, bosentan in combination with atorvastatin exerted an additive effect in attenuating the progression of atherosclerosis. This suggests the potential use of endothelin receptor antagonists in combination with lipid-lowering drugs for anti-atherosclerotic therapy to address pathogenetic factors beyond LDL-C, such as inflammation [111]. De Haro *et al*. [112, 113] evaluated the use of bosentan in the treatment of peripheral artery disease, which can be a manifestation of ASCVD. In a randomized controlled trial, bosentan treatment improved absolute claudication distance, ankle–brachial index, and plasma C-reactive protein levels. During a 3-year follow-up period of bosentan treatment, the risk of major adverse cardiovascular events was significantly reduced compared with placebo [112, 113]. To summarize, the existing literature on ET-1 targeting in atherosclerosis is scarce; however, the results of the aforementioned studies are promising and warrant further research. Fig. 1 demonstrates the involvement of ET-1 in the pathogenesis of atherosclerosis.

### 2.4 Pulmonary Hypertension

Pulmonary hypertension (PH) is a condition with abnormally high pressure in the pulmonary artery. Many abnormalities, including HF and chronic obstructive pulmonary disease (COPD), can cause pulmonary arterial hypertension PAH. The diagnosis is made by catheterizing the right heart; a pressure greater than 20 mmHg is considered hypertension. Subsequently, patients are categorized as precapillary, postcapillary, or combined PH. Combining this information with clinical background allows patients to be grouped into appropriate clinical groups associated with potential causative factors. This classification aims to introduce more individualized treatment methods depending on the clinical group of PAH patients [114]. Pathogenesis of PH involves dysfunction of the endothelium and VSMCs. The endothelium releases fewer vasodilators, such as NO and prostacyclin, and promotes the secretion of vasoconstrictors, such as ET-1. Consequently, there is an overgrowth of smooth muscles and a reduction in vascular diameter. A prolonged condition leads to impaired cardiac function and RV HF. An extensive number of echocardiographic parameters are altered in patients with PH, including an enlarged right ventricle, an enlarged right atrial area, a distended inferior vena cava, or the presence of pericardial effusion, demonstrating a significant impact of the disease on the functionality of the right heart [115].

**Fig. 2.**
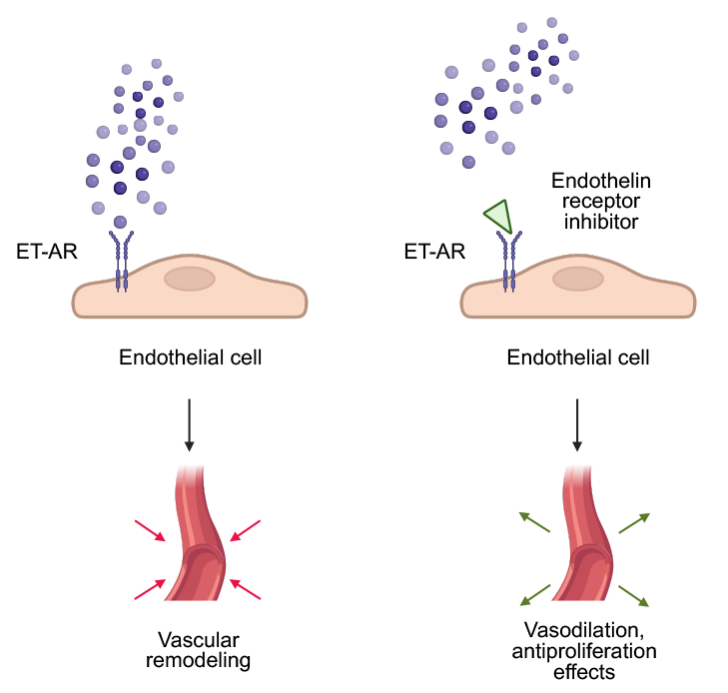
**The role of ET-1 and its inhibitors in pulmonary hypertension**. Created in BioRender. Physiology, D. (2025) https://BioRender.com/i0xwrir.

Treatment of PH can be either interventional or pharmacological. The first group greatly depends on the subtype of hypertension. Treatment can involve pulmonary artery denervation or even lung transplantation in patients resistant to current treatment methods [116, 117]. Pharmacology offers several options for treating PAH. These options highlight the varying effectiveness of drugs across clinical groups of patients with PH. Phosphodiesterase 5 inhibitors, sildenafil and tadalafil, act by blocking the disintegration of cyclic guanosine monophosphate, a vasodilator. Agents from this group are associated with greater efficacy in patients with COPD and PH. In contrast, no efficacy was observed in a clinical trial including patients with HF [118, 119]. Other agents used in the treatment of PH include calcium channel blockers, prostacyclin agonists, and sotatercept, a recently approved TGF-β ligand trap [114, 120]. Bosentan, ambrisentan, and macitentan are used among the agents that target endothelins. These agents prevent and reverse pulmonary vasoconstriction and pulmonary vascular remodeling, thereby improving symptoms and exercise capacity and reducing morbidity and mortality (Fig. 2) [121, 122]. Bosentan, the first dual ET-1 inhibitor approved in 1995, is associated with hepatotoxicity and numerous drug interactions [123]. Moreover, bosentan is also teratogenic and causes headaches, flushing, and edema in the lower limbs [82]. Bosentan has antioxidative properties; in PH mouse models, this drug reduced levels of advanced oxidation protein products and increased the ratio of reduced glutathione to oxidized glutathione [124]. Bosentan also showed cardioprotective features in preclinical models of chemotherapy-induced cardiotoxicity [125].

Ambrisentan exhibits a similar efficacy but fewer drug interactions and a lower risk of hepatic injury compared with bosentan [126]. However, ambrisentan also causes headaches, peripheral edema, dizziness, and upper respiratory system infections [122]. Macitentan, in turn, is the most potent dual inhibitor of these three agents. Macitentan has a longer duration of action and higher antagonistic potency in pulmonary smooth muscle cells, as investigated in the SERAPHIN study [121]. Therefore, macitentan significantly improves 6-minute walking distance and reduces mortality [127]. Moreover, macitentan causes fewer adverse effects, the most common being nasopharyngitis, headache, and anemia [128]. Compared to other agents, macitentan has a lower potential of promoting the development of peripheral edema and hepatic injury [121].

Researchers continue to search for proper combinations or treatment strategies to increase the efficacy of ET-1 inhibitors in PH. For instance, a preclinical study evaluated the potential of inhaled bosental microparticles [129]. Meanwhile, administering a new drug to rats elicited positive feedback [130]: the agent reduced pulmonary artery systolic deceleration time and right ventricular internal diameter compared with animals in the positive control groups, suggesting promising results and translational potential. Other advancements include self-nanoemulsifying drug delivery systems and the design of novel derivatives [131, 132]. Another method to improve the efficacy of ET-1 antagonists is through appropriate combinations. Indeed, treatment with bosentan and sacubitril/valsartan demonstrated improved hemodynamic properties in the pulmonary circulation and normalized cardiac remodeling [133]. Bosentan use was recently studied in patients with chronic thromboembolic pulmonary hypertension (CTEPH). In those patients with severe preoperative PH, the use of bosentan as a bridge to the procedure was associated with a significantly higher decrease in pulmonary vascular resistance compared to patients without bridging medication [134]. Nevertheless, the guidelines state that preoperative use remains controversial, with the efficacy of endothelin receptor antagonists in such settings and delays in referral for surgery [115].

Ambrisentan is another ET-1 inhibitor approved for the treatment of PH. Years after approval, studies have continued to support the positive effects of ambrisentan in patients with PH. Indeed, Lan *et al*. [135] analyzed the initial use of ambrisentan combined with tadalafil in patients with severe PH. Notably, the use of this strategy for 6 months was associated with improvements in functional status, the 6-minute walk test, NT-proBNP concentrations, and pulmonary arterial pressure and right ventricular parameters. Few studies have examined the efficacy resulting from the transition from bosentan to ambrisentan. Published results appear controversial, suggesting a possible influence of age on the response to the switch. In adults, the transition was safe and efficacious; however, apart from the left ventricular end-diastolic dimension, no other significant echocardiographic differences were observed [136]. In contrast, in a pediatric population, a switch from bosentan with sildenafil to ambrisentan with tadalafil was associated with several improvements in functional status and echocardiography [137].

Currently, clinical researchers are focused on identifying biomarkers associated with response in various fields of medicine. Hatano and colleagues [138] analyzed these biomarkers in relation to the reaction to ambrisentan. The researchers demonstrated that female sex, the use of aldosterone antagonists, baseline NT-proBNP, and the 6-minute walking test were associated with a response to treatment.

Macitentan is the third ET receptor inhibitor approved for the treatment of PH. A recent meta-analysis by Du and Yuan [139] analyzed 11 clinical trials and observational studies to summarize the efficacy of the drug in patients with PH. Pooled analyses demonstrated that the drug effectively reduced pulmonary vascular resistance, cardiac index, and NT-proBNP levels. Conversely, the meta-analysis did not find significant results in other hemodynamic parameters, such as the 6-minute walking test or mPAP.

Recently, extensive investigations were conducted to assess the efficacy of macitentan across various combinations and settings in patients with PH. For instance, McLaughlin *et al*. [140] investigated the potential use of combination therapy with macitentan and tadalafil in patients with cardiac comorbidities. The authors aimed to assess the role of these drugs in the cohort recommended for treatment with a monotherapy strategy, as outlined in the 2022 guidelines. The authors analyzed the results of the TRITON and REPAIR clinical trials and observed a similar safety profile and efficacy of the combination across groups with and without cardiac comorbidities.

In an interesting analysis by Paoli and colleagues [141], the researchers evaluated a large cohort of PH patients treated with macitentan or other endothelin receptor antagonists. In the latter group, the majority of patients received ambrisentan, while only a few received bosentan. The authors retrospectively analyzed 897 patients, among whom 518 received macitentan and 379 received other agents. Macitentan use was associated with a significantly lower risk of hospitalization and intensive care unit stays compared with the other cohort. These observations highlight the potential of macitentan to reduce hospitalization-related healthcare costs. Nevertheless, regarding the cost analysis, macitentan is more expensive than bosentan, but provides a better quality of life, which further proves the appropriate cost-effectiveness of the therapy [142].

### 2.5 Heart Failure

The number of studies investigating the role of endothelin in HF is constantly increasing. Currently, the effect of ET-1 in HF is equivocal owing to the different receptors involved. ET-A activation promotes myocardial inflammatory injury, while ET-B has an opposite influence [143]. In HF patients, the level of ET-1 is increased, and ET-A and ET-B are activated, thus vasoconstriction is promoted [144]. In turn, endothelial-derived NO-mediated vasodilation is attenuated because of endothelial dysfunction induced by excess ET-1 [143]. These processes contribute to cardiac dysfunction. In addition, ET-1 promotes collagen accumulation, resulting in cardiac fibrosis, which impairs diastole [145]. ET-1 also attenuates collagenase activity, leading to cardiac hypertrophy [146].

In patients with HF and reduced ejection fraction, higher ET-1 levels were correlated with worse clinical outcomes, including lower ejection fraction, eGFR, functional status, and laboratory parameters [147]. Macitentan, a dual inhibitor, alleviates collagen deposition and decreases cardiomyocyte size [144]. Furthermore, macitentan attenuates oxidative stress and reduces both systolic and diastolic BP. Altogether, Macitentan treatment leads to improved cardiac relaxation and increased myocardial perfusion [148]. Unfortunately, recently published results of the SERENADE trial demonstrated no benefit of macitentan in patients with HF with preserved or mildly reduced ejection fraction [149]. Sitaxsentan, a selective ET-A receptor blocker, does not alter cardiomyocyte structure nor improve diastolic function [144].

## 3. Limitations of Endothelin-1 Antagonists: A Focus on Fluid Retention

Fluid retention is a well-known side effect of ERA therapy. In the PRECISION trial, fluid retention was the most common adverse event, which typically developed within the first 4 weeks of aprocitentan administration. The severity was mainly mild to moderate, and management included diuretic treatment. Naturally, the complication was observed more often in the subgroup with chronic kidney disease [73]. Regarding ET-A inhibition, fluid retention was suggested to result from an adaptive response of venodilation and increased venous capacity [150]. In a study including patients after subarachnoid hemorrhage, age was proposed as an associated factor with fluid retention [151]. Furthermore, the simultaneous use of vasospasm fasudil hydrochloride also constituted a risk factor for the development of fluid retention in this population [152]. Thus, understanding the pathogenesis of fluid retention should be accompanied by management strategies for this complication.

Initial post hoc data from the SONAR study suggested that combining atrasentan with an SGLT2 inhibitor could mitigate fluid retention, as evidenced by a smaller increase in body weight and B-type natriuretic peptide (BNP) levels, alongside a greater reduction in albuminuria compared with atrasentan alone [153]. However, higher zibotentan doses (5 mg), even when combined with dapagliflozin, still led to fluid retention, resulting in early discontinuation of those arms [154]. This highlights a dose-dependent risk of fluid retention with ERA therapy that might be attenuated (but not eliminated) by SGLT2 inhibitors. These findings support the potential of low-dose selective ERA–SGLT2i combination therapy as a promising strategy. Ongoing Phase III trials, such as ZENITH High-Proteinuria, aim to determine whether the decrease in albumin levels in response to zibotentan and dapagliflozin will have a lasting effect on kidney protection [155]. A randomized, double-annonymized, placebo-controlled Phase IIa/b trial is currently evaluating the efficacy of combining zibotentan with the inhibitor dapagliflozin in patients with portal hypertension, a condition with a high risk of fluid retention. The addition of dapagliflozin aims to counteract the fluid retention by enhancing diuresis and natriuresis. Outcomes will be compared to the dapagliflozin and placebo groups [156].

## 4. Emerging Areas in Other Conditions

In the previous sections, this review has focused on CVDs, in which several ET-1 inhibitors have been examined and registered. However, extensive investigations are also studying the role of ET-1 in other diseases. For instance, ET-1 was suggested to contribute to the pathogenesis of metabolic diseases; stimulation of mouse adipocytes with ET-1 was shown to downregulate the insulin-sensitizing adiponectin [157]. This activity is mediated by ET-B, as ET-B receptor knockout improved insulin and glucose tolerance [157]. Furthermore, ET-1 has a role in the dysfunction of perivascular adipose tissue observed in obesity. ET-1 was identified as contributing to reduced Nrf2 activity and increased ROS [158]. Additionally, positive correlations between ET-1 levels and waist and hip circumferences were recently observed [159].

Malignancies represent another group of diseases with accumulating studies investigating the role of ET-1. Such studies highlight the potential of using ET-1 for diagnostic purposes, as demonstrated by Irfan *et al*. [160]. Researchers have shown that salivary ET-1 levels were increased in patients with premalignant and oral cancer lesions compared to healthy controls [160]. These findings suggest that monitoring ET-1 could become a screening method for cancer in the future. Nihei and colleagues [161] also evaluated the potential use of ET-1 as a biomarker. The authors demonstrated that monitoring ET-1 in patients treated with the antiangiogenic bevacizumab (a monoclonal antibody targeting vascular endothelial growth factor) could identify those with ≥ grade 2 proteinuria, a possible adverse event of bevacizumab [161]. Additionally, targeting ET-1 was also evaluated as a potential anticancer strategy. In tumoroid ovarian cancer models, macitentan suppressed the stroma-invasive properties of cancer cells [162], demonstrating translational potential for future investigations.

## 5. Conclusions

ET-1 and its receptors significantly contribute to endothelial health by regulating vascular tone. Mechanisms leading to CVDs frequently involve dysregulation of endothelial functionality by promoting dysfunction and inflammation. Current evidence demonstrates that ET-1 plays a role in the pathogenesis of endothelial dysfunction by mediating vasoconstriction, impairing vasodilation, promoting vascular remodeling, and contributing to inflammation. These properties indicate that ET-1 contributes to the pathophysiology of CVD, prompting preclinical and clinical investigations to determine whether suppressing ET-1 activity could be clinically useful. The result of these studies is the approval of aprocitentan in the treatment of arterial hypertension based on the results of the PRECISION clinical trial. The broadening of potential drugs used in the treatment of hypertension is a major finding; however, more trials are required to address the efficacy of aprocitentan in combination with other classic antihypertensive medications and the effectiveness of these combinations against classic agents. Regarding future studies, Professor Allan S. Brett highlights the need to evaluate the efficacy of these drugs in patients with higher BP levels in long-term settings, as well as the role of aprocitentan at the individual level [163].

The use of ET-1 inhibitors is well established in clinical practice in patients with PH, reflected in comprehensive clinical guidelines. Nevertheless, ongoing efforts are increasing the effectiveness of these inhibitors by designing novel drug formulations and identifying the most effective therapeutic combinations. Furthermore, the availability of several drugs in this family allows for switching between therapeutics. However, more clinical trials are needed to evaluate whether the transition between ET-1 inhibitors is efficacious and safe. Furthermore, a complex analysis of biomarkers is required to identify patients who are more likely to respond to a particular ET-1 inhibitor. More experiments are expected as engineered pulmonary artery tissues develop, enabling disease modeling and drug testing [164]. However, fewer studies have investigated the potential of targeting ET-1 in conditions such as atherosclerosis or HF, underscoring the need for further research to provide a more comprehensive understanding of the role of ET-1 inhibitors.
